# Geriatric Consultation Reduces High-risk Medication Usage at Discharge in Elderly Trauma Patients

**DOI:** 10.7759/cureus.3649

**Published:** 2018-11-28

**Authors:** Jyoti Sharma, Manisha Parulekar, Peter Stewart, Melissa Blatt, Tania Zielonka, Themba Nyirenda, Christopher Rogers, Lisa Tank

**Affiliations:** 1 Surgery, Hackensack University Medical Center, Hackensack, USA; 2 Internal Medicine, Hackensack Univeristy Medical Center, Hackensack, USA; 3 Miscellaneous, Hackensack University Medical Center, Hackensack, USA; 4 Internal Medicine, Hackensack University Medical Center, Hackensack, USA

**Keywords:** delirium, geriatric trauma, geriatric consultation, high risk medications

## Abstract

Background

Traumatic injury in a growing geriatric population is associated with higher mortality and complication rates. Geriatric consultation (GC) is vital in reducing risk factors that contribute to adverse outcomes. This study aims to determine if receiving a GC had an impact on high-risk medication usage.

Methods

Patients eligible for a GC, age ≥ 65, and length of stay > two days, were identified via a chart review from July 2013 to July 2014 at a Level II trauma center. This population was divided into those with and without a GC. Data collected included demographics, injury severity, medications, delirium, mortality, and readmissions. High-risk medications were defined using the Beers Criteria. Statistical analysis involved using appropriate standard tests to compare groups, including multivariate logistic regression.

Results

Forty-nine of a total of 104 patients received a GC. Groups were comparable on injury severity score, co-morbidities, and high-risk medication use upon admissions. The GC group was 74% less likely to be discharged on high-risk medications than the non-GC group.

Conclusion

GC in elderly trauma patients reduces high-risk medication use upon discharge. Further studies are needed to explore how GC impacts readmission rates and mortality. A multidisciplinary trauma team, including a geriatrician, must exist to address the unique medical, psychological, functional, and social issues of a growing, aged trauma population.

## Introduction

In 2014, 46 million (15.0%) or about one in every seven individuals were age 65 and older; however, by 2060, this is expected to more than double to 98 million (1). Moreover, persons age 85 and older are estimated to grow significantly from 6 million in 2014 to 14.6 million in 2040 [1]. With a growing geriatric population, the incidence of traumatic injury will also increase [2]. As of 2013, unintentional injury was the eighth leading cause of death in the 65 and older population [3]. One in three older adults falls each year, leading to direct medical costs totaling $34 billion in 2013 [4]. This cost, along with the total number of falls, is projected to increase significantly as the United States population continues to age [4].

High-risk medications, such as benzodiazepines, sedatives, and psychotropic drugs in elderly patients, have been associated with an increased risk of falls and delirium [5]. In a systematic review and meta-analysis, Leipzig, Cumming, and Tinetti [6] found that elderly patients who were prescribed psychotropic, benzodiazepine, or sedative medications had higher odds of falling. The use of benzodiazepines, both in the inpatient as well as the outpatient setting, has been associated with adverse outcomes in the elderly such as increased sedation, decreased attention, anterograde amnesia, falls with associated fractures, hemorrhage with associated hypotension, hypoglycemic encephalopathy, and liver failure [7-11].

Trauma injury in the elderly is also associated with higher mortality and complication rates as compared to younger patients [2]. The management of an older population requires a multidisciplinary approach that takes into consideration the decreased physical reserve and the presence of multiple comorbidities in these patients [2]. Multiple studies have shown that a proactive geriatric consultation (GC) alone or within a formal geriatric protocol has been linked with fewer episodes of delirium, fewer in-hospital falls, less likelihood of discharge to a long-term care facility, and a shorter length of stay [2,5,12-14]. According to 22 randomized trials with greater than 10,000 subjects, those who received a GC followed by appropriate medical care were 25% more likely to be alive and in their own home at one year after discharge [2,12-13].

A geriatrician’s participation in medication reconciliation, pain management, disposition decisions, and advance care planning effectively reduces in-hospital complications in older individuals [12-13]. Few studies have examined the effect of GC on the use of polypharmacy at discharge. This study aims to determine if receiving a GC had an impact on high-risk medication use at discharge in elderly trauma patients.

## Materials and methods

A retrospective study was performed at Hackensack University Medical Center (HUMC), which is a level II trauma center with 775 beds and approximately 900 traumas per year. The institutional trauma registry was queried for all subjects age 65 and older from July 2013 to July 2014. Variables collected from the chart included demographics (age, gender, height, weight, body mass index (BMI), race, ethnicity), level of activation, mechanism of injury, admission GCS, injury severity score (ISS), co-morbidities, initial vital signs, the presence of delirium, hospital medications, the presence of a GC, and high-risk medication use (i.e., benzodiazepines, opiates, and sedatives). Primary outcomes included the length of stay (LOS), discharge medications, discharge locations, readmission rates, and in-hospital mortality. Composite variables were created and included the following: depression (i.e., the patient had a diagnosis of depression and/or was using medications for depression) and multi-traumatic brain injury (TBI) (one or more anatomical region of hemorrhage as indicated by computed tomography (CT) of the head).

**Figure 1:**
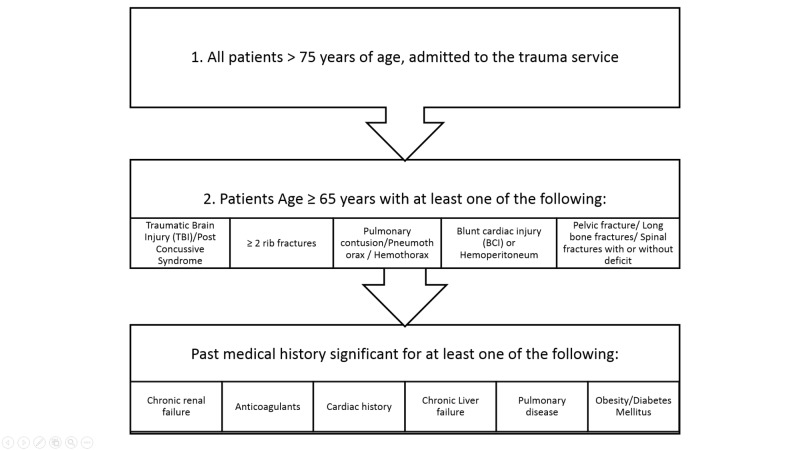
Criteria for Requesting a Geriatric Consult on Trauma Patients

The HUMC department of trauma has developed a protocol based on American College of Surgeons (ACS) Trauma Quality Improvement Program (TQIP) Geriatric Trauma Management Guidelines [2] to determine if geriatricians should be consulted at admission (Figure 1). This protocol highlighted trauma patient criteria that should prompt a GC, but the ultimate decision was left to the discretion of the trauma surgeon. Patients were excluded if they did not meet the criteria for a GC, if expected LOS was less than 48 hours, and if patients were in a moribund condition or died in the trauma bay. Geriatricians, in conjunction with the trauma team, provided a multidisciplinary patient-centered approach with a specific focus on pharmacokinetic, social, physiological, behavioral, and somatic health factors.

Delirium was defined using the five key features from the Diagnostic and Statistical Manual of Mental Disorders, 5th Edition (DSM-V) [15], which include a disturbance: (1) in attention and awareness, (2) that develops over a short time period and is a shift from patient’s baseline and fluctuates throughout the day, (3) in cognition, (4) that cannot be attributed to other previously existing conditions or disorders, and (5) cannot be accounted for by a medical condition, substance abuse/withdrawal, or medication side effect. Furthermore, for this retrospective analysis, the key components of delirium were identified via documentation of the following in the electronic medical record (EMR): positive confusion assessment method (CAM) scores [16], use of restraints/mitts, sitter, and confusion/agitation in the chart or delirium noted in the patient’s problem list.

In this study, high-risk medications were defined using the Beers criteria [17]. The Beers criteria (2) provides guidelines for safe and effective medication use while minimizing adverse drug events for the geriatric population. The criteria itself is a comprehensive medication list published elsewhere [17]. The variable “high-risk geriatric meds” included the following five categories of medications: anti-depressants, antipsychotics, benzodiazepines, sedatives, and opioids. Polypharmacy was defined as the use of more than five medications of any type at admission or discharge.

Descriptive analyses were conducted as follows: categorical/discrete variables were summarized as frequency (%), continuous variables were reported as mean ± standard deviation (SD) or median with interquartile range (IQR: 25th - 75th percentile), depending on whether the data were normally distributed. The assumption of the normality of the data distribution was assessed using the Shapiro-Wilk test. A comparison of categorical variables between groups was conducted using the Pearson chi-square or Fisher’s exact test, as appropriate. A comparison of continuous variables between groups was performed using a two-sided t-test for normally distributed data or Wilcoxon rank sum test for non-normally distributed data. Further, for a comparison of continuous variables between more than two groups, one-way analysis of variance (ANOVA) or the Kruskal-Wallis test, as appropriate, was conducted.

Univariate logistic regression analyses were performed to identify associations between variables. All of the variables that were significantly associated with being discharged on high-risk medications were entered in a multivariable model in which the stepwise selection procedure was employed to obtain a final model fit. Modeled covariates in univariate and multivariate logistic regression analyses were reported as odds ratio (OR) with 95% confidence intervals (CI). A p-value < 0.05 was chosen as the cut-off level for statistical significance. All data analysis was performed using SAS™ (version 9.4; SAS Institute Inc., Cary, NC, US).

Approval for the study was obtained from the HUMC institutional review board.

## Results

A total of 104 charts were reviewed and met the criteria for geriatric consultation. Of these, 49 patients received a GC and 55 did not. Table 1 compares the demographic variables of both groups. Patients who received a GC were older (81.0 vs. 83.0 year old, p = 0.041) and had a lower BMI (24.6 vs. 27.3 kg/m2, p = 0.017) compared to those without. The GC group did appear to have slightly worse injuries in regards to injury severity score (9 vs. 10) although this finding was not statistically significant (p = 0.052). Approximately70% of patients in both groups had three or more co-morbidities. However, patients in the non-GC group were more likely to have pre-existing depression compared to those with GC (27.3% vs. 10.2%, p = 0.046). Patients with a GC were more likely to be on an anticoagulant than those without (26.5% vs. 10.9%, p = 0.040). Table 1 also highlights delirium in the non-GC and GC groups. Most patients had a diagnosis of delirium prior to a GC. Patients with a GC and delirium were more likely to have restraints ordered (p = 0.026), documentation of a CAM positive score on the step-down unit (p = 0.017), and sitter usage (p = 0.017) as compared to the non-GC group.

**Table 1:** Demographic Characteristics of Patients With and Without a Geriatric Consultation (N=104) BMI, body mass index; MVA, Motor vehicle accident; GCS, Glasgow coma scale; ISS, Injury severity score; SICU, Surgical intensive critical care unit; TBI, Traumatic brain injury; CHF, Congestive heart disease; CAD, Coronary artery disease; HTN, Hypertension; CVA, Cardiovascular accident; GC, Geriatric consultation; CAM, Confusion assessment method; ICU, Intensive care unit; DNR, Do not resuscitate; DNI, Do not intubate * Statistically significant, p < 0.05, **Values may not add up to the total N due to missing variables

Variables	Non-GC (N=55)	GC (N=49)	P-Value
Age, Median (25^th^-75^th^), Years	81.1 (70.0-87.0)	83.0 (77.0-90.0)	0.041*
Gender Female, n (%)	34 (61.8)	28 (57.1)	0.691
Race			0.237
White	49 (89.0)	43 (87.8)	
Black	1 (1.8)	4 (8.1)	
Asian	5 (9.0)	2 (4.1)	
Ethnicity			0.185
Non-Hispanic	52 (94.5)	42 (85.7)	
Hispanic	3 (5.5)	7 (14.3)	
BMI, median (25^th^ – 75^th^), kg/m^2^	27.3 (24.3 – 30.6)	24.6 (21.4 – 28.9)	0.017*
Trauma Activation, N (%)			0.592
Level I	2 (3.6)	3 (6.1)	
Level II	34 (61.8)	25 (51.0)	
Consult	19 (34.6)	15 (42.9)	
Mechanism of Injury, N (%)			0.286
MVA	11.0 (20.0)	6 (12.2)	
Fall	44.0 (80.0)	43.0 (87.8)	
Admission GCS, Median (25^th ^– 75^th^)	15.0 (15.0-15.0)	15.0 (15.0-15.0)	0.227
ISS, Median (25^th ^– 75^th^)	9.0 (5.0-12.0)	10 (8.0-14.0)	0.052
SICU Admission, n (%)	17 (30.9)	22 (44.9)	0.160
Intubation, n (%)	6 (10.9)	5 (10.2)	1.000
Injury, n (%)			
TBI	22 (40.0)	23 (46.9)	0.554
Major Fracture	27 (49.1)	26 (53.1)	0.700
Minor Fracture	16 (29.6)	17 (34.7)	0.674
Comorbidities, n (%)			
CHF	9 (16.4)	7 (14.3)	0.769
CAD	18 (32.7)	17 (34.7)	0.832
Cardiac Arrhythmia	18 (32.7)	15 (30.6)	0.817
HTN	48 (87.3)	39(79.6)	0.305
CVA	8 (14.6)	4 (8.2)	0.369
Diabetes Mellitus	17 (30.9)	14 (28.6)	0.795
Lung Disease/COPD/Asthma	7 (12.7)	8 (16.3)	0.602
Liver Disease	1 (1.8)	1 (2.0)	1.000
Kidney Disease	2 (3.6)	2 (4.1)	1.000
Depression	15 (27.3)	5 (10.2)	0.046*
Dementia	8 (14.6)	12 (24.5)	0.199
Parkinson	2 (3.6)	4 (8.2)	0.417
Alcoholism	5 (9.1)	0 (0.0)	0.059
Psychosis	0 (0.0)	1 (2.0)	0.476
Co-Morbidities ≥ 3, n (%)	40 (72.7)	39 (79.6)	0.414
Anticoagulant Use, n (%)	6 (10.9)	13 (26.5)	0.040*
Delirium, n (%)			
Delirium, all	24 (43.6)	28 (57.1)	0.169
At Admission	10 (18.2)	13 (26.5)	0.306
Prior to GC	N/A	24 (50.0)	N/A
Delirium Identifiers, n (%)			
Restraints Ordered	7 (12.7)	15 (30.6)	0.026*
Mitts Ordered	7 (12.7)	8 (18.4)	0.426
CAM Positive – Step Down Unit	11 (20.0)	20 (41.7)	0.017*
CAM Positive – ICU **	4 (23.5)	8 (36.4)	0.494
Sitter Usage	5 (9.1)	13 (27.1)	0.017*
Delirium on Problem List	15 (27.8)	19 (40.4)	0.180
Agitation	16 (29.6)	13 (27.1)	0.776
Code Status on Discharge, n (%)			0.104
Full code	26 (76.5)	21 (58.3)	
DNR	6 (17.7)	14 (38.9)	
DNR/DNI	2 (5.9)	1 (2.8)	

The non-GC and GC groups had comparable LOS, in-hospital mortality, and 30-day readmission (Table 2). The patients who received a GC were more likely to have a urinary tract infection (UTI) compared to the non-GC patients (20.4% vs. 5.5%, p = 0.035). More patients in the non-GC group were likely to be discharged to acute rehabilitation than the GC patients; however, this was not statistically significant.

**Table 2:** Outcomes Variables in Elderly Trauma Patients With and Without GC LOS, Length of stay; ICU, Intensive care unit; UTI, Urinary tract infection; DVT, Deep vein thrombosis; SNF, Skilled nursing facility * Statistically significant p < 0.05 **Values may not add up to the total N due to missing variables

Variables	Non-GC (N=55)	GC (N=49)	P - value
LOS, Median (25^th ^– 75^th^), Days	5.0 (4.0-8.0)	5.0 (3.0-10.0)	0.908
ICU LOS, Median (25^th ^– 75^th^)	0.0 (0.0-1.0)	0.0 (0.0-2.0)	0.162
In-hospital Mortality, n (%)	3 (5.5)	5 (10.4)	0.468
Morbidity, n (%)			
Pneumonia	5 (9.1)	3 (6.1)	0.720
UTI	3 (5.5)	10 (20.4)	0.035*
DVT	4 (7.3)	2 (4.1)	0.682
Renal Failure	1 (1.8)	3 (6.1)	0.341
Discharge Location, n (%)			0.178
Home	10 (18.2)	14 (28.6)	0.248
SNF	14 (25.5)	14 (28.6)	0.826
Acute Rehab	27 (49.1)	16 (32.7)	0.112
Readmission 30-days, n (%) **	6 (11.5)	4 (9.1)	0.746

Table 3 highlights the high-risk medication status of geriatric trauma patients with and without a GC at three different time periods: at admission, in hospital, and at discharge. Both groups at admission had comparable uses of high-risk medications, but non-GC patients had a statistically significant increased usage of benzodiazepines and opioids. Despite the fact that patients in both the non-GC and the GC groups have a similar use of high-risk medications while in the hospital, the GC group was less likely to be discharged on high-risk geriatric medications compared to the non-GC group (73.1% vs. 47.7%, p = 0.011). Lower rates of benzodiazepine and sedative use were also noted at discharge in the GC group (26.9% vs. 4.4%, p = 0.003; 17.3% vs. 0.0%, p = 0.003) versus the non-GC group.

**Table 3:** Medication Status in Geriatric Trauma Patients With and Without a Geriatric Consultation * Statistically significant p < 0.05 **Values may not add up to the total N due to missing variables

Variables	Non-GC (N=55)	GC (N=49)	P-value
At Admission			
Medication Type, n (%)			
Anti-Depressants	17 (30.9)	15 (30.6)	0.974
Anti-Psychotics	4 (7.3)	2 (4.1)	0.682
Benzodiazepines	11 (20.0)	3 (6.1)	0.047*
Sedatives	3 (5.5)	1 (2.0)	0.620
Opioids	11 (20.0)	2 (4.1)	0.017*
Polypharmacy (>5meds), n (%)	37 (67.3)	30 (61.2)	0.520
High-Risk Geriatric Meds, n (%)	29 (52.7)	21 (42.9)	0.315
In Hospital			
Medication Type, n (%)			
Anti-Depressants	15 (27.3)	14 (28.6)	0.883
Anti-Psychotics	10 (18.2)	14 (28.6)	0.209
Benzodiazepines	25 (45.5)	14 (28.6)	0.076
Sedatives	16 (29.1)	14 (28.6)	0.954
Opioids	42 (76.4)	39 (79.6)	0.692
High-Risk Geriatric Meds, n (%)	50 (90.9)	45 (91.8)	1.000
At Discharge **			
Medication Type, n (%)			
Anti-Depressants	15 (28.9)	12 (26.7)	0.811
Anti-Psychotics	4 (7.7)	2 (4.4)	0.683
Benzodiazepines	14 (26.9)	2 (4.4)	0.003*
Sedatives	9 (17.3)	0 (0.0)	0.003*
Opioids	26 (50.0)	15 (33.3)	0.096
Polypharmacy (>5meds), n (%)	44 (84.6)	42 (95.5)	0.103
High-Risk Geriatric Meds, n (%)	38 (73.1)	21 (47.7)	0.011*

The univariate logistic analysis (Table 4) indicated that there was a statistically significant association between being discharged on high-risk medications and the following variables: BMI (OR = 1.12), BMI overweight vs. normal (OR = 3.83), BMI obese vs. normal (OR = 5.70), GC (OR = 0.34), anti-depressant use on admission (OR = 5.66), high-risk medication use on admission (OR = 5.32), anti-depressant use during hospitalization (OR = 26.47), depression (OR = 6.06), multi-TBI (OR = 0.23), subarachnoid hemorrhage (OR = 0.33), and the presence of a major fracture (OR = 2.69). The final multivariable analysis model (Table 4) indicated that GC, depression, and multi-TBI were significantly and independently associated with being discharged on high-risk medications. The odds of being discharged on high-risk medications for patients who received GC were 74% lower than for patients who did not receive a GC (OR = 0.26, 95% CI: 0.10 to 0.68, P = 0.006). Having depression was associated with a seven-fold increased odds of being discharged on high-risk medications as compared to those not having depression (OR = 7.07, 95% CI: 2.02 to 24.73, p = 0.002). Patients with multi-TBI were also approximately 90% less likely to be discharged on high-risk medications than those without (OR = 0.11, 95% CI: 0.02 to 0.65, p = 0.015).

**Table 4:** Univariate and Multivariate Predictors of High-risk Medication Use at Discharge BMI, body mass index; GC, Geriatric consultation; TBI, Traumatic brain injury; OR, Odds ratio; CI, Confidence interval * Statistically significant p < 0.05

Variable	Univariable			Multivariable		
	OR	95% CI	P	OR	95% CI	P-value
BMI	1.136	1.037 – 1.245	0.006*			
BMI, Categories			0.012*			
Underweight vs. Normal	1.500	0.189 – 11.927	0.702			
Overweight vs. Normal	3.833	1.375 – 10.687	0.010*			
Obese vs. Normal	5.700	1.726 – 18.828	0.004*			
GC vs. Non-GC	0.336	0.144 – 0.788	0.012*	0.255	0.096 – 0.680	0.006*
Medications at Admission						
Anti-Depressants	5.656	1.772 – 18.047	0.003*			
Anti-Psychotics	1.273	0.221 – 7.320	0.787			
Benzodiazepines	2.597	0.673 – 10.019	0.166			
Sedatives						
Opioids	4.010	0.836 – 19.241	0.083			
High-Risk Geriatric Meds	5.232	2.090 – 13.101	<0.001*			
Medications During Hospitalization						
Anti-Depressants	26.470	3.397 – 206.236	0.002*			
Anti-Psychotic	1.333	0.482 – 3.691	0.580			
Benzodiazepines	2.127	0.874 – 5.174	0.096			
Sedatives	0.920	0.362 – 2.34	0.862			
Opioids	1.846	0.705 – 4.833	0.212			
High-Risk Geriatric Meds	13.526	1.590 – 115.048	0.017*			
Depression	6.064	1.903 – 19.323	0.002*	7.068	2.020 – 24.725	0.002*
Traumatic Injury						
TBI	0.524	0.227 – 1.208	0.129			
Multi-TBI	0.230	0.055 – 0.953	0.043*	0.111	0.019 – 0.647	0.015*
Subdural Hemorrhage	0.735	0.291 – 1.856	0.515			
Subarachnoid Hemorrhage	0.327	0.119 – 0.901	0.031*			
Intraventricular Hemorrhage	0.621	0.038 – 10.236	0.739			
Intraparenchymal Hemorrhage	0.614	0.083 – 4.558	0.634			
Concussion	0.600	0.257 – 1.398	0.237			
Major Fracture	2.692	1.149 – 6.310	0.023*			

## Discussion

An early-implemented, proactive multidisciplinary trauma-geriatric model has consistently shown in the literature to prevent and successfully manage geriatric syndromes (i.e., delirium), preserve function, and facilitate discharge planning [12-13,18]. This study further supports this model by highlighting a significant difference (74%) in high-risk medication use, specifically due to the lower utilization of benzodiazepines and sedatives upon discharge in the hospitalized elderly trauma patient. This difference was not seen in the GC and non-GC groups while hospitalized. One possibility for this findings is that the in-hospital high-risk medication use is a snapshot in time, i.e., the temporal pathway of high-risk medication use is unclear. Likely, the “at-discharge” time point reflects both a discontinuation and a reduction of high-risk medication use during the hospitalization and at the time of discharge.

Few studies have specifically assessed the impact of GC on high-risk medication prescriptions in the trauma population. However, there has been extensive research on high-risk medication use and its impact on falls and delirium [19]. The side effects of benzodiazepine use are well-established in both inpatient and outpatient settings in the geriatric population [7-10]. Brief interventions in the primary care setting have led to a significant reduction in benzodiazepine use in the elderly [20]. In the hospital, early proactive geriatric involvement in the care of an elderly trauma patient includes a comprehensive geriatric assessment (CGA). This multidimensional, multidisciplinary diagnostic instrument is designed to collect data on medical, psychosocial, and functional capabilities and the limitations of elderly patients, which aids in developing treatment and follow-up plans [2].

In this study, although the GC group had more frail patients (older, lower BMI, and slightly higher ISS), the readmission and LOS rates were similar in both groups. Moreover, these patients were more likely to go home (although this was not statistically significant). Many studies have looked at the positive impact of home discharge versus a skilled nursing facility (SNF) or an acute rehab facility on the medical, psychological, functional, and social state of elderly patients [21]. The positive impact of home care on elderly health outcomes includes the prevention of unplanned hospitalizations, a reduction in the number of hospital days, improvement in cognitive health, increased functional abilities, and improvements in quality of life [22-24]. This research suggests that a multidisciplinary trauma team that includes a geriatrician can increase the likelihood that elderly patients will be discharged home rather than to an SNF or acute rehab facility, which has positive implications for the medical and social health of these patients.

A large portion of the study population had a diagnosis of delirium prior to GC, limiting the examination of how GC affected this important syndrome. Consistent with prior literature, this study demonstrated that hyperactive delirium was more prevalent (i.e., documentation indicating the use of restraints/mitts, sitter, confusion/agitation, CAM scores, etc.) and more often diagnosed as well as treated than its counterpart, hypoactive delirium, in patients who received a GC [25]. The treatment of hypoactive and hyperactive delirium varies significantly. Diagnosing hypoactive delirium in older patients is key to ensure the apt execution of acute nonpharmacological treatment strategies such as reorientation and behavioral intervention [20]. Additionally, the timely diagnosis of hyperactive delirium is important so that appropriate pharmacological therapy can be administered to preserve patient safety [20]. In hyperactive delirium, inappropriate high-risk medication use, such as benzodiazepines, can worsen the condition and ultimately lead to increased mortality [2]. The long-term effects of delirium have been well-documented in the literature and include an increased risk of institutionalization, cognitive, and functional decline as well as mortality, especially with intensive care unit admission [26-28]. The etiology of delirium is multifactorial, involving genetics, direct brain insults (i.e. metabolic, inflammatory, and/or neurotransmitter abnormalities), and an aberrant stress response [25,29]. All three types, hyperactive, hypoactive, and mixed, of delirium can occur during a single hospitalization. The complex pathogenesis of delirium requires a multidisciplinary approach with the geriatrician for prevention, early detection, and management, especially in older patients.

**Figure 2:**
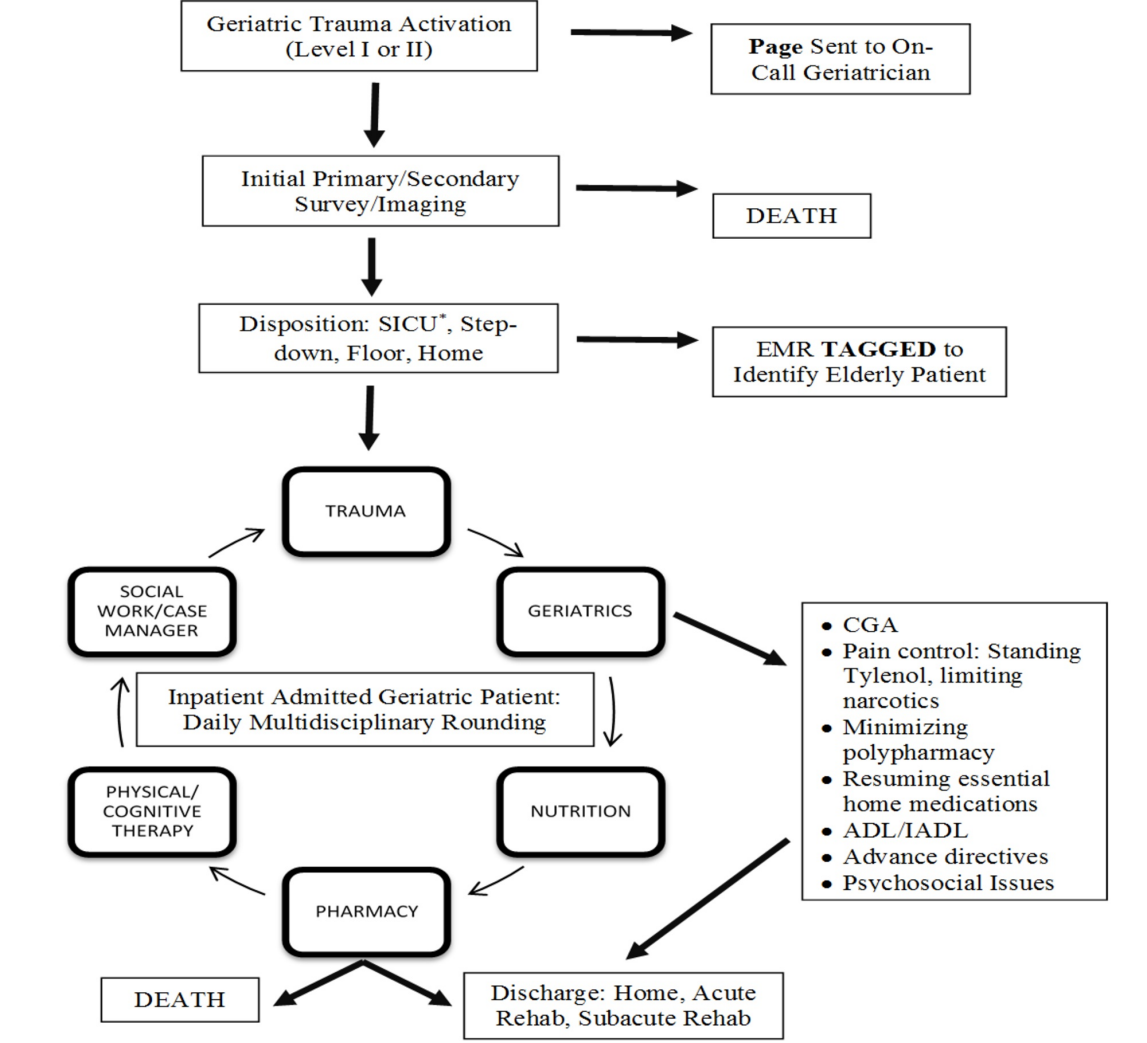
Algorithm for a Multidisciplinary Team Approach to a Hospitalized Geriatric Trauma Patient SICU, Surgical critical care unit; EMR, Electronic medical record; CGA, Comprehensive geriatric assessment; ADL, Activities of daily living; IADL, Instrumental activities of daily living

A trauma-geriatric collaborative effort can change the culture of prescribing high-risk medications in the inpatient environment among a vulnerable population. In our institution, the standard of care for geriatric trauma patients has evolved to include the following: using low doses of high potency medications, attempting to prohibit benzodiazepine use unless clinically warranted, ordering standing intravenous or by-mouth acetaminophen, frequent reorientations, slow weaning of standing pre-hospitalization high-risk medications (i.e. benzodiazepines), and active family involvement. Since this retrospective study, efforts have increased at our institution to enforce and standardize the criteria for a GC request (Figure 1). Elderly trauma patients with delirium on admission or the onset of delirium during hospitalization should also prompt a GC, a criterion that will likely need to be added to our protocol (Figure 1, Number 2). Understandably, the latter will aid with delirium management, but not prevention. Moreover, the timing of GC has been also enforced at HUMC to within 48 hours of patient admission since this study showed that most elderly patients were already delirious at the time of consultation. Furthermore, Figure 2 highlights the projected seamless flow of care of an elderly trauma patient from the emergency room (ER) to discharge. The geriatric trauma population is unique and requires constant vigilance, starting in the ER by flagging the electronic medical record (EMR) and the early involvement of the multidisciplinary team for rapid recovery and discharge.

There are several limitations to this study. First, this was a single-center retrospective study with a relatively small sample size and included only geriatric trauma patients. Thus, these results cannot be generalized to other populations or trauma centers. Second, this research was unable to determine if GC affected delirium incidence since a significant number of patients were already delirious prior to consultation. The selection bias of obtaining a GC likely affects the result of this study, i.e., most trauma surgeons obtained a GC in high-risk elderly patients (those with signs and symptoms of delirium). Furthermore, in cases where geriatricians are involved, there is likely actual heightened awareness of the primary providers that may be contributing to the overall decrease in the number of high-risk medications during the hospital course or at discharge. Additionally, this study did not track non-pharmacological interventions (i.e. physical therapy, family presence, hospital elder life program) for delirium [30]. This type of intervention may have contributed to the overall decrease in high-risk medication use at discharge in the GC group. Also, the reliability of the retrospective data extracted from the EMR is limited due to the possibility of poor clinical documentation. Lastly, there is no post-hospital follow-up to confirm that the discontinuation and/or avoidance of high-risk medications was persistent over a long period of time. There is also no data collected about post-hospital functional and cognitive outcomes. Further data will be needed to establish whether these changes are lasting and concomitant with behavioral changes in prescribing the high-risk medications.

Future studies should examine the impact of GC on-discharge medication reconciliation and post-discharge outcomes. The gold standard continues to be a prospective assessment of delirium and high-risk drug prescription use in the geriatric trauma population. Furthermore, extending this collaborative model to other services, such as neurosurgery, orthopedics, and general surgery, may reduce inappropriate high-risk medication use in the elderly.

## Conclusions

As the population ages, a larger number of older patients are requiring hospital care due to traumatic injury. These patients are at a higher risk of delirium, leading to an increase in morbidity and mortality as compared to younger patients. This study further identifies that a GC reduces high-risk medication use upon discharge. A multidisciplinary trauma team, including a geriatrician, addresses the unique medical, psychological, functional, and social issues of this population.
